# Stromal Cell-Derived Factor-1*α* Alleviates Calcium-Sensing Receptor Activation-Mediated Ischemia/Reperfusion Injury by Inhibiting Caspase-3/Caspase-9-Induced Cell Apoptosis in Rat Free Flaps

**DOI:** 10.1155/2018/8945850

**Published:** 2018-01-11

**Authors:** Li Song, Li-Na Gao, Jun Wang, Swosti Thapa, Yong Li, Xiao-Bo Zhong, Hong-Wei Zhao, Xue-Rong Xiang, Fu-Gui Zhang, Ping Ji

**Affiliations:** ^1^Chongqing Key Laboratory for Oral Diseases and Biomedical Sciences, Chongqing 401147, China; ^2^Department of Oral and Maxillofacial Surgery, Stomatological Hospital of Chongqing Medical University, Chongqing 401147, China; ^3^Chongqing Municipal Key Laboratory of Oral Biomedical Engineering of Higher Education, Chongqing 401147, China

## Abstract

Surgical flaps are frequently affected by ischemia/reperfusion (I/R) injury. Calcium-sensing receptor (CaSR) and stromal cell-derived factor-1*α* (SDF-1*α*) are closely associated with myocardial I/R injury. This study was performed to evaluate the feasibility of applying SDF-1*α* to counteract CaSR activation-mediated I/R injury in ischemic free flaps. Free flaps that underwent ischemia for 3 h were equally randomized into five groups: CaCl_2_, NPS2143 + CaCl_2_, SDF-1*α* + CaCl_2_, AMD3100 + SDF-1*α* + CaCl_2_, and normal saline. The free flaps were harvested to evaluate flap necrosis and neovascularization after 2 h or 7 d of reperfusion. p-CaSR/CaSR was extensively expressed in vascular endothelial cells of free flaps after I/R injury, and activation of the SDF-1*α*/CXCR4 axis and NPS2143 could reduce the expression of cleaved caspase-3, caspase-9, FAS, Cyt-c, and Bax and increase Bcl-2 expression; the opposite was true after CaSR activation. Interestingly, initiation of the SDF-1*α*/CXCR4 axis might abrogate CaSR activation-induced I/R injury through enhancement of microvessel density. In conclusion, CaSR might become a novel therapeutic target of free flaps affected by I/R injury. Activation of the SDF-1*α*/CXCR4 axis and NPS2143 could counteract CaSR activation-mediated I/R injury and promote free flap survival through inhibition of caspase-3/caspase-9-related cell apoptosis and enhancement of neovascularization.

## 1. Introduction

Surgical flaps are frequently used to repair defects caused by congenital diseases, trauma, or tumor excisions in plastic and reconstructive surgery. However, partial or total necrosis of flaps after early ischemia is commonly encountered [1, 2]. Ischemia/reperfusion (I/R) injury is considered the primary cause of flap necrosis [3].

Calcium-sensing receptor (CaSR), which was originally cloned from parathyroid chief cells by Brown et al. in 1993 [4], is expressed in various tissues and organs such as the myocardium, brain, lung, and kidney [5, 6]. Increasing evidence has demonstrated that CaSR is associated with cell apoptosis and “calcium overload,” causing I/R injury [7, 8]. Recent studies have suggested that CaSR is also expressed in vascular smooth muscle cells [9], human umbilical vein endothelial cells [10], porcine coronary artery endothelial cells [11], and the rabbit superior mesenteric artery [12], but not in the vascular endothelial cells of skin flaps.

Stromal cell-derived factor-1 (SDF-1), which belongs to the CXC chemokine family, was initially characterized as a pre-B-cell stimulatory factor and cloned from mouse bone marrow stromal cells by Nagasawa et al. in 1994 [13]. SDF-1 is well known for its promotional function in neovascularization, including angiogenesis and vasculogenesis. For example, SDF-1 plays a critical role in angiogenesis by regulating endothelial sprouting in vitro and in vivo [14]; this can significantly reduce ischemic free flap necrosis [15, 16], as we previously reported [17]. SDF-1 also confers protection against myocardial I/R injury with less apoptotic cell death [18]. However, no previous studies have shown either protection against I/R injury or an antiapoptotic effect by SDF-1 in skin flaps.

This study was performed to determine whether CaSR is functionally expressed in the vascular endothelial cells of free flaps and plays a critical role in I/R injury of free flaps. The authors also assessed whether the administration of SDF-1*α* can protect free flaps against I/R injury and alleviate CaSR activation-mediated I/R injury in rat free flaps. Finally, whether the above effects can be neutralized by the CXCR4 antagonist AMD3100 was also examined.

## 2. Materials and Methods

### 2.1. Establishment of Oversized Epigastric Axial Skin Flap Model to Mimic Free Flap Model

This study was approved by the Chongqing Medical University Medical Center Institutional Animal Care and Use Committee. Male Sprague-Dawley rats (Central Animal Laboratory of Chongqing Medical University) weighing 220 to 280 g were housed under specific pathogen-free conditions and treated according to National Institutes of Health guidelines. Specialists at Chongqing Medical University monitored the health of the animals weekly. The rats were anesthetized by intraperitoneal injection of sodium pentobarbital (30 mg/kg) as previously described [19]. The right superficial epigastric artery- and vein-based epigastric axial skin flaps were approximately 5 × 6 cm in size and comprised skin and subcutaneous fascia, including the panniculus carnosus, as previously described [17], while the opposite superficial epigastric vessels were ligated [20]. All rats were equally randomized into Groups A, B, C, D, and E (*n* = 10 per group), in which they received CaCl_2_ (0.1 mL/kg; Sigma-Aldrich, St. Louis, MO, USA), NPS2143 (1 mg/kg; Selleck Chemicals, Houston, TX, USA) + CaCl_2_, SDF-1*α* (10 mg/kg; Cyagen Biosciences, Santa Clara, CA, USA) + CaCl_2_, AMD3100 (5 mg/kg; Sigma-Aldrich) + SDF-1*α* + CaCl_2_, and normal saline (NS) only, respectively [21]. The second reagents were administrated through the femoral vein 10 min after the first treatment and 10 min earlier than the third treatment. A 3 h period of flap ischemia was elected among different time points (i.e., hours 1, 2, 3, and 4) based on the highest expression of phosphorylated CaSR (p-CaSR) protein (Supplemental Figure 1). Upon completion of a 3 h period of ischemia induced by clamping the pedicle vessels with double microvascular clamps [22] to mimic ischemia of free flaps, the clamps were removed, and all of the free flaps were sutured to the original sites with interrupted 5-0 Ethilon (Ethicon, Inc., Somerville, NJ, USA). The rats recovered from anesthesia in a 20°C to 25°C environment before they were returned their feeding cages, and penicillin (200,000 U/d) was intramuscularly administered for 3 d thereafter. The rats' diet, mental status, and flap survival were observed and recorded daily. The free flaps were harvested after 2 h or 7 d of reperfusion (*n* = 5 per group), and all rats were killed by an overdose of sodium pentobarbital.

### 2.2. Evaluation of Free Flap Survival

The flaps were photographed on postoperative day 7. The survival status of the free flaps was blindly assessed with respect to their color, presence of bleeding upon cutting, gross appearance, and area, even if covered with scar tissue. The area of survival and total flap surface area were evaluated using Image-Pro Plus software (version 5.0; Media Cybernetics LP, Silver Spring, MD, USA). The necrotic ratio was expressed as the percentage of necrotic area relative to the total flap surface area as previously described [17]. The tissues harvested from the conjunctive areas between the flaps and the recipients were cut into 5 *μ*m thick paraffin sections or prepared for other assays.

### 2.3. Western Blot Analysis

Western blot analysis was conducted as previously described [17, 23]. Briefly, tissues from the conjunctive area were collected, lysed in modified RIPA buffer, centrifuged, and quantified using the Bradford method (Beyotime, Shanghai, China). After quantification of the protein concentration, equal amounts of protein lysate were separated by sodium dodecyl sulfate-polyacrylamide gel electrophoresis according to established protocols. The proteins were transferred from the gels to PVDF membranes (Pall, Port Washington, NY, USA) in a sandwich model at 200 mA for 90 min. The membranes were then placed in TBS/T, probed with antibodies to p-CaSR (1 : 500; Bioworld Technology, St. Louis Park, MN, USA), CXCR4 (1 : 200; Santa Cruz Biotechnology, Dallas, TX, USA), Cyt-c (1 : 1000; Cell Signaling Technology, Danvers, MA, USA), Bax (1 : 100; Santa Cruz Biotechnology), Bcl-2 (1 : 200; Santa Cruz Biotechnology), caspase-9 (1 : 200; Santa Cruz Biotechnology), FAS (1 : 200; Santa Cruz Biotechnology), cleaved caspase-3 (1 : 1000; Cell Signaling Technology), or *β*-actin (1 : 1000; Cell Signaling Technology), and incubated at 4°C overnight. All membranes were followed by anti-rabbit/anti-mouse secondary antibodies (1 : 1000; Beyotime) at 37°C for 2 h. Quantity One (Version 4.5.2; Bio-Rad, Hercules, CA, USA) was used to determine the protein expression, which was recorded as the ratio of the target protein relative to *β*-actin [24].

### 2.4. Quantitative Real-Time PCR (qPCR) Analysis

qPCR was performed to detect the mRNA expression of a variety of signaling pathways, such as CaSR, CXCR-4, Cyt-c, and FAS, as previously described [23, 25]. Total RNA was isolated from the conjunctive areas using TRIZOL Reagent (Invitrogen, Thermo Fisher Scientific, Waltham, MA, USA) and subjected to reverse transcription reactions with hexamer and M-MuLV reverse transcriptase (New England Biolabs, Ipswich, MA, USA). These cDNA products were used as the PCR templates. The primer sequences used in real-time qPCR were as follows: CaSR forward 5′-TGGCTCCCTGATCGGCTATACC-3′, reverse 5′-GGGAAGGCTTGAAGAGGATAATGTA-3′; CXCR4 forward 5′-GCAATGGGTTGGTAATCCTG-3′, reverse 5′-CCAGAAGGGGAGTGTGATGA-3′; Cyt-c forward 5′-CACAGATGCCAACAAGAACAA-3′, reverse 5′-GTCTGCCCTTTCTCCCTTCT-3′; FAS forward 5′-GTCTTGGGGATTTGCCTACA-3′, reverse 5′-GAACGCTACTGGGTTTGTCC-3′; and GAPDH forward 5′-GACATGCCGCCTGGAGAAAC-3′, reverse 5′-AGCCCAGGATGCCCTTTAGT-3′. PCR was performed using a real-time qPCR system (SYBR® Premix Ex Taq™ II; TaKaRa, Tokyo, Japan), with 39 cycles of denaturation at 95°C for 30 s, annealing at 60°C for 30 s, and polymerization at 72°C for 30 s. The relative gene expression levels were calculated according to ΔCt (ΔΔCt) method as follows: target amount = 2^−ΔΔCt^ [26]. GAPDH was used as an internal control.

### 2.5. Immunohistochemical (IHC) Analysis and Neovascularization

IHC was used to detect the types of vital molecules expressed in tissues from conjunctive areas collected after 2 h or 7 d of reperfusion as previously described [17, 25, 27]. Briefly, 5 *μ*m thick paraffin sections were stained with antibodies to CD34 (1 : 250; Abcam, Cambridge, UK) and CaSR (1 : 200; Bioworld Technology). The microvessel density (MVD) in neovascularization was assessed by measuring the number of CD34-positive capillaries in 18 fields as described by Hollingsworth et al. [28].

### 2.6. Apoptosis Assay

An In Situ Cell Death Detection Kit (Roche, Basel, Switzerland) was utilized for quantitative detection of apoptotic cells by labeling DNA strand breaks (free 3′-OH terminal) at the single-cell level [29]. Briefly, 5 *μ*m thick paraffin sections were managed as in regular IHC, and then TUNEL reaction mixture (TdT-mediated dUTP-X nick-end labeling, at 37°C for 60 min), Converter-POD (at 37°C for 30 min), and DAB (at room temperature for 10 min) were sequentially added to the sections. The apoptotic ratio was calculated as the percentage of the number of TUNEL-positive cells relative to the total number of vascular endothelial cells [30].

### 2.7. Statistical Analysis

All data are expressed as the mean ± standard deviation and were evaluated using SPSS (Version 19.0; IBM Corp., Armonk, NY, USA). Comparisons were examined using Tukey's method for one-way analysis of variance. A value of *P* < 0.05 was considered statistically significant. The histograms were created by GraphPad Prism 5 (GraphPad Software, La Jolla, CA, USA), and the final figures were assembled by CorelDRAW(R) Graphics Suite X4 (Corel, Ottawa, Ontario, Canada).

## 3. Results

### 3.1. Evaluation of Free Flap Survival

**Figure 1:**
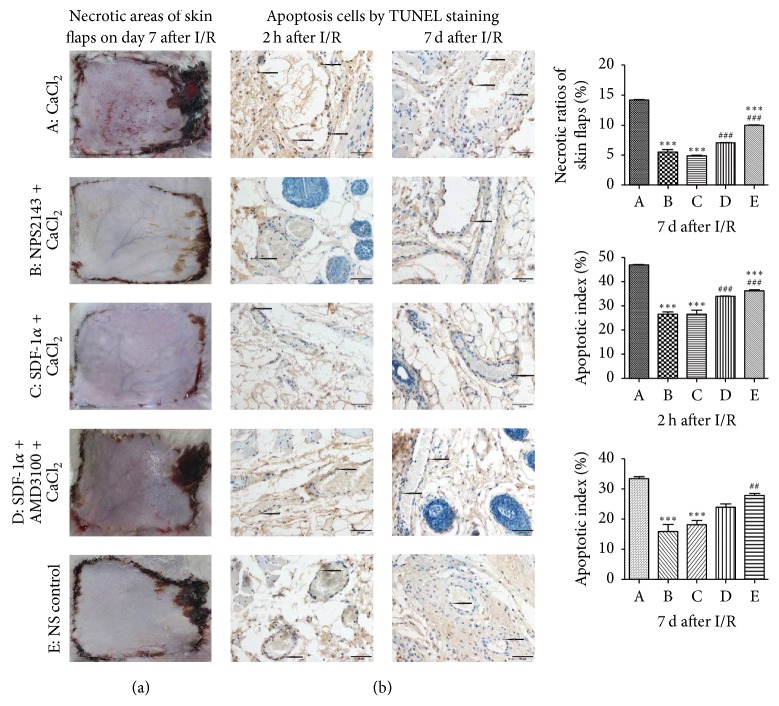
(a) Free flap survival status on day 7 after ischemia/reperfusion (I/R). The necrotic ratio in the CaCl_2_ group was significantly higher than that of the other groups, while the necrotic ratios in the NPS2143 + CaCl_2_ and SDF-1*α* + CaCl_2_ groups were significantly lower than that in the NS group. (b) Apoptotic cell analysis by TUNEL staining after 2 h or 7 d of reperfusion. Apoptotic cells in the CaCl_2_ group were remarkably increased, while apoptotic cells in the NPS2143 + CaCl_2_ and SDF-1*α* + CaCl_2_ groups were notably decreased compared with the other groups. ^*∗∗∗*^*P* < 0.001 versus CaCl_2_ group, ^###^*P* < 0.001 versus SDF-1*α* + CaCl_2_ group, ^##^*P* < 0.01 versus SDF-1*α* + CaCl_2_ group.

The necrotic ratio (percentage of necrotic area relative to total flap surface area) was calculated by computer-aided planimetry to evaluate the free flap survival on postoperative day 7. Five deciduous flaps were supplemented because of scratching by the rats. As shown in Figure 1(a), the necrotic sites were consistently located at the edge and distal end of each flap. The necrotic ratios in the five groups (CaCl_2_, NPS2143 + CaCl_2_, SDF-1*α* + CaCl_2_, AMD3100 + SDF-1*α* + CaCl_2_, and NS) were 14.16%  ±  0.36%, 5.49%  ±  0.62%, 4.87%  ±  0.24%, 7.00%  ±  0.09%, and 9.92%  ±  0.41%, respectively. The necrotic ratio in the CaCl_2_ group was significantly higher than those in the NS control group and other treatment groups, while the necrotic ratios in the NPS2143 + CaCl_2_ and SDF-1*α* + CaCl_2_ groups were significantly lower than that in the NS group (Figure 1).

### 3.2. Apoptosis Analysis

As shown in Figure 1(b), apoptotic cells were detected by TUNEL staining after 2 h or 7 d of reperfusion. The numbers of apoptotic cells in the CaCl_2_ group were remarkably higher than those in the other groups, while the apoptotic cells in the NPS2143 + CaCl_2_ and SDF-1*α* + CaCl_2_ groups were notably decreased (Figure 1).

### 3.3. Immunohistochemical Analysis and Assessment of Neovascularization

**Figure 2:**
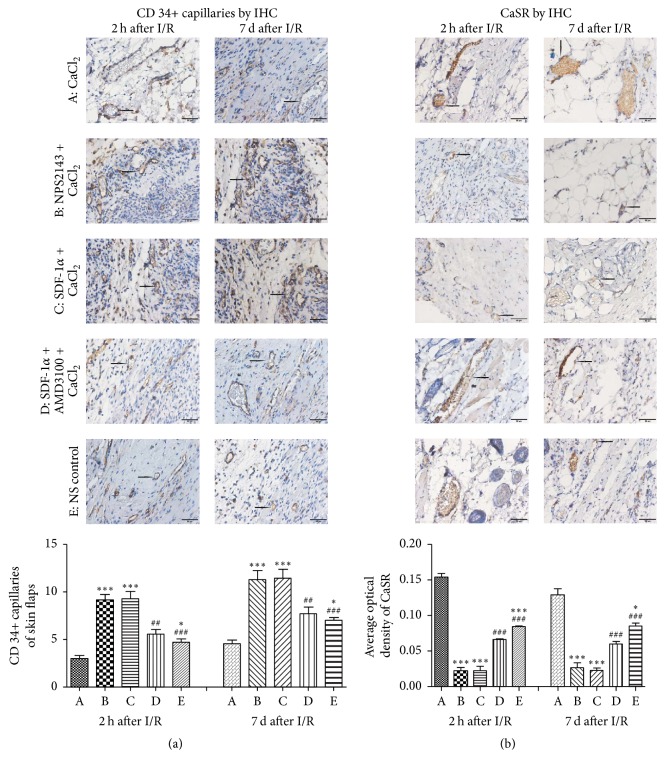
(a) MVD in neovascularization stained by CD34-positive capillaries was examined using IHC staining after 2 h or 7 d of reperfusion. The CaCl_2_ group yielded the lowest MVD, while the NPS2143 + CaCl_2_ and SDF-1*α* + CaCl_2_ groups exhibited notably enhanced neovascularization compared with the control group. (b) CaSR by IHC staining after 2 h or 7 d of reperfusion. The CaCl_2_ group exhibited the highest expression of CaSR in vascular endothelial cells and partial blood cells, while the expressions levels of CaSR in the NPS2143 + CaCl_2_ and SDF-1*α* + CaCl_2_ groups were lower than those in the NS control group. ^*∗∗∗*^*P* < 0.001 versus CaCl_2_ group, ^*∗*^*P* < 0.05 versus CaCl_2_ group, ^###^*P* < 0.001 versus SDF-1*α* + CaCl_2_ group, ^##^*P* < 0.01 versus SDF-1*α* + CaCl_2_ group.

The MVD was evaluated to assess the neovascularization of free flaps affected by I/R injury after 2 h or 7 d of reperfusion (Figure 2(a)). The mean MVDs for the five groups after 2 h of reperfusion were 3.00 ± 0.81, 9.14 ± 1.57, 9.29 ± 1.98, 5.57 ± 1.27, and 4.71 ± 0.95, while the mean MVDs for the five groups after 7 d of reperfusion were 4.57 ± 0.98, 11.29 ± 2.56, 11.43 ± 2.51, 7.71 ± 1.80, and 7.00 ± 0.82. These groups acquired the same tendency at two different points. The CaCl_2_ group showed the lowest MVD, while the NPS2143 + CaCl_2_ and SDF-1*α* + CaCl_2_ groups showed notably enhanced neovascularization compared with the control group (Figure 2).

As shown in Figure 2(b), CaSR was assessed by IHC staining. The mean optical densities of the five groups after 2 h of reperfusion following ischemia were 0.154 ± 0.005, 0.022 ± 0.004, 0.022 ± 0.006, 0.066 ± 0.002, and 0.084 ± 0.002, while the mean optical densities after 7 d of reperfusion were 0.129 ± 0.009, 0.026 ± 0.007, 0.023 ± 0.003, 0.060 ± 0.004, and 0.085 ± 0.004; that is, these groups also manifested the same trend. The CaCl_2_ group exhibited the highest expression of CaSR in vascular endothelial cells and partial blood cells, while the NPS2143 + CaCl_2_ and SDF-1*α* + CaCl_2_ groups showed the lowest CaSR expression compared with the NS control group (Figure 2).

### 3.4. Expressions of Various Proteins by Western Blot Analysis

**Figure 3:**
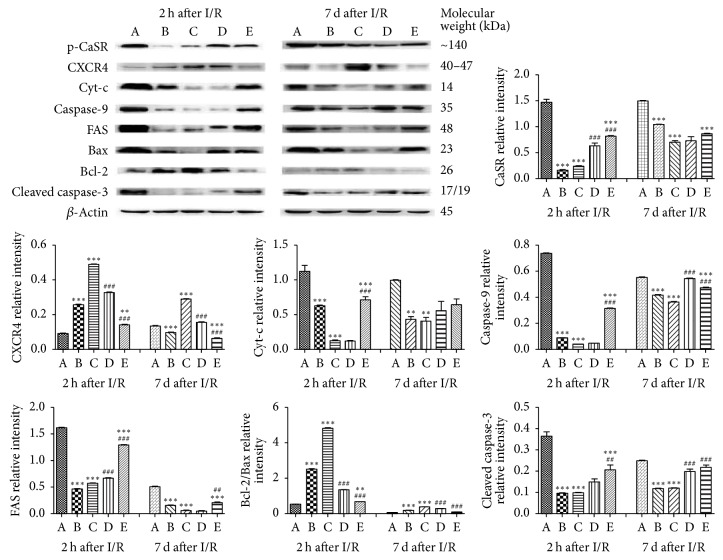
The protein expression levels of p-CaSR, CXCR4, FAS, Cyt-c, caspase-9, Bax, Bcl-2, and cleaved caspase-3 were assessed by western blot analysis after 2 h or 7 d of reperfusion. The expression levels of p-CaSR, FAS, Cyt-c, caspase-9, and Bax were highest in the CaCl_2_ group, while the expression levels of these proteins were significantly lower in the NPS2143 + CaCl_2_ and SDF-1*α* + CaCl_2_ groups. The expression levels of p-CaSR showed a significant difference at hour 2 but not on day 7 after I/R of free flaps. The expression level of CXCR4 was the strongest while that of caspase-9 was the weakest in the SDF-1*α* + CaCl_2_ group over time. ^*∗∗∗*^*P* < 0.001 versus CaCl_2_ group, ^*∗∗*^*P* < 0.01 versus CaCl_2_ group, ^###^*P* < 0.001 versus SDF-1*α* + CaCl_2_ group, ^##^*P* < 0.01 versus SDF-1*α* + CaCl_2_ group.

The protein expression levels of p-CaSR, CXCR4, FAS, Cyt-c, caspase-9, cleaved caspase-3, Bax, and Bcl-2 were assessed by western blot analysis after 2 h or 7 d of reperfusion of free flaps. The expression levels of p-CaSR, FAS, Cyt-c, caspase-9, cleaved caspase-3, and Bax were higher in the CaCl_2_ group than in all other groups, while these proteins showed significantly lower expression levels in the NPS2143 + CaCl_2_ and SDF-1*α* + CaCl_2_ groups. The expression level of p-CaSR was significantly different after 2 h of reperfusion between the different treatment groups after I/R of free flaps; however, no significance difference was observed after 7 d of reperfusion among Groups B, C, D, and E after I/R. The expression levels of FAS and Cyt-c showed the same tendency both after 2 h and 7 d of reperfusion of free flaps. The protective protein CXCR4 showed the strongest expression, while the proapoptotic protein caspase-9 displayed the weakest expression with time in the SDF-1*α* + CaCl_2_ group. Unlike the other proapoptotic proteins, Bcl-2/Bax showed higher expression in the SDF-1*α* + CaCl_2_ group than in all other groups (Figure 3).

### 3.5. mRNA Expressions by qPCR Analysis

**Figure 4:**
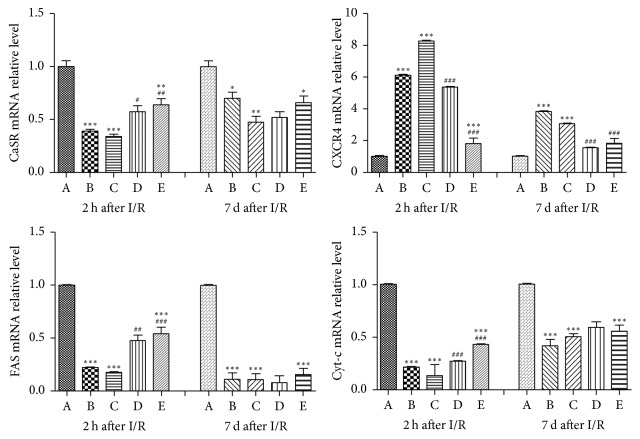
The mRNA expression levels of CaSR, CXCR4, FAS, and Cyt-c were detected by qPCR analysis after 2 h or 7 d of reperfusion of free flaps. The expression levels of CaSR, FAS, and Cyt-c were higher in the CaCl_2_ group than those in all other groups at both time points. The expression level of CXCR4 in the SDF-1*α* + CaCl_2_ group was significantly higher than that in the CaCl_2_ and control groups after I/R of free flaps. ^*∗∗∗*^*P* < 0.001 versus CaCl_2_ group, ^*∗∗*^*P* < 0.01 versus CaCl_2_ group, ^*∗*^*P* < 0.05 versus CaCl_2_ group, ^###^*P* < 0.001 versus SDF-1*α* + CaCl_2_ group, ^##^*P* < 0.01 versus SDF-1*α* + CaCl_2_ group, ^#^*P* < 0.05 versus SDF-1*α* + CaCl_2_ group.

The mRNA expression levels of CaSR, CXCR4, FAS, and Cyt-c were detected by qPCR analysis after 2 h or 7 d of reperfusion of free flaps. The expression level of CaSR was higher in the CaCl_2_ group than those in the NPS2143 + CaCl_2_ and SDF-1*α* + CaCl_2_ groups at both time points. The expression level of CXCR4 in the SDF-1*α* + CaCl_2_ group was significantly higher than that in the CaCl_2_ group and control group after I/R. Furthermore, the expression levels of FAS and Cyt-c were higher in the CaCl_2_ group than those in all other groups after 2 h or 7 d of reperfusion of free flaps, showing a consistent tendency with the expression levels of CaSR and western blot analysis (Figure 4).

## 4. Discussion 

In this study, the authors investigated whether CaSR is functionally expressed in the vascular endothelial cells of free flaps and plays a detrimental role in I/R injury of free flaps. They also assessed whether administration of SDF-1*α* can protect free flaps from I/R injury and alleviate CaSR partial (control group) or extensive (such as CaCl_2_ group) activation-mediated I/R injury in rat free flaps.

According to the results from the CaCl_2_ (a CaSR agonist) group, NPS2143 (a CaSR inhibitor) + CaCl_2_ group, SDF-1*α* (a CXCR4 agonist) + CaCl_2_ group, AMD3100 (a CXCR4 antagonist) + SDF-1*α* + CaCl_2_ group, and NS control group, p-CaSR and CaSR were extensively expressed in the vascular endothelial cells of free flaps (Figures 2 and 3). Additionally, the level of p-CaSR expression was closely associated with the level of I/R injury.

Functional studies have demonstrated that activation of CaSR by I/R could lead to myocardial I/R injury by activating cell apoptosis [6, 31, 32] and “calcium overload” because CaCl_2_ is a typical agonist of CaSR [7]; however, NPS2143 is known to inhibit CaSR [33]. Consistent with other studies, the authors demonstrated that a high expression level of p-CaSR indicates severe I/R injury of free flaps, while a low p-CaSR expression level indicates mild I/R injury (Figures 1–4). The CaCl_2_ group exhibited significantly more flap necrosis, which was consistent with the higher number of apoptotic cells detected by the TUNEL staining (Figure 1); lower MVD and more CaSR detected by IHC staining (Figure 2); the highest proapoptotic protein expressions of p-CaSR, cleaved caspase-3, and caspase-9 detected by western blot analysis (Figure 3); and the highest mRNA expression of CaSR detected by qPCR analysis (Figure 4). However, the NPS2143 + CaCl_2_ group exhibited less flap necrosis, which was consistent with fewer apoptotic cells (Figure 1), a high MVD, less CaSR (Figure 2), and low proapoptotic protein expressions of p-CaSR, cleaved caspase-3 and caspase-9 (Figure 3) after 2 h of reperfusion but not always after 7 d of reperfusion of free flaps. These results imply that the potential hypoxic signaling pathways that stimulate revascularization were downregulated and that the revascularization process might cease after 7 days [34]. These results suggest that extensive CaSR activation plays a detrimental role in I/R injury of free flaps by promoting caspase-3/caspase-9-dominated cell apoptosis and that extensive activation of CaSR could be induced by the administration of CaCl_2_ while this proapoptotic effect could be neutralized by NPS2143_._

A variety of approaches or reagents have been administered to exert preventive effects against necrosis of free flaps or skin grafts [1, 17, 35–37]. According to previous studies, SDF-1 plays a critical and unique role in angiogenesis and vasculogenesis by enhancing MVD in neovascularization [15, 17]. AMD3100 functions as an antagonist of CXCR4 [16]. No relationship has previously been observed between CaSR and the SDF-1*α*/CXCR4 biological axis. However, the present study initially revealed that the SDF-1*α*/CXCR4 axis was intimately associated with CaSR activation in I/R injury of free flaps. The SDF-1*α* + CaCl_2_ group exhibited the least flap necrosis, which was consistent with fewer apoptotic cells (Figure 1), high MVD, less CaSR (Figure 2), and low proapoptotic protein expression of p-CaSR, cleaved caspase-3 and caspase-9 (Figure 3) after 2 h of reperfusion but not necessarily after 7 d of reperfusion of free flaps, revealing a tendency very similar to that in the NPS2143 (i.e., a CaSR inhibitor) + CaCl_2_ group. The AMD3100 + SDF-1*α* + CaCl_2_ group displayed an almost opposite tendency compared with the SDF-1*α* + CaCl_2_ group. These results suggest that the SDF-1*α*/CXCR4 axis activated by SDF-1*α* might protect free flaps from I/R injury by inhibiting caspase-3/caspase-9-induced cell apoptosis and that the activation of SDF-1*α*/CXCR4 axis and this antiapoptotic effect could almost be neutralized by AMD3100_._

**Figure 5:**
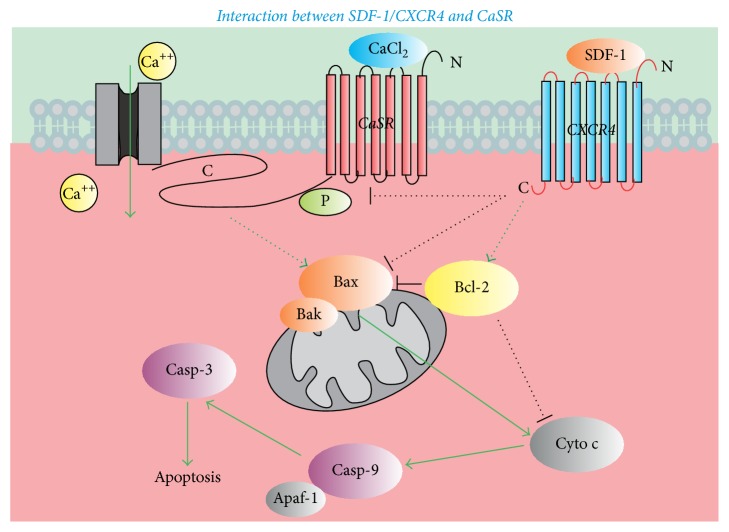
Schematic diagram of the interaction between SDF-1*α*/CXCR4 and CaSR. Activation of CaSR increased the expression of Bax, then Cyt-c, caspase-9, and cleaved caspase-3; it finally caused cell apoptosis. Activation of the SDF-1*α*/CXCR4 axis alleviated this process as Bcl-2.

These results provide insight into the activation of the SDF-1*α*/CXCR4 axis, showing that this axis could function as a CaSR inhibitor to significantly alleviate the extensive CaSR activation-mediated I/R injury of free flaps through the inhibition of cell apoptosis by reducing the proapoptotic protein expressions of FAS, Bax, Cyt-c, caspase-9, and cleaved caspase-3 and increasing the antiapoptotic protein expression of Bcl-2 (Figure 5). Interestingly, these results also suggest that initiation of the SDF-1*α*/CXCR4 axis might neutralize the CaSR partial or extensive activation to alleviate the I/R injury of free flaps by enhancement of MVD in neovascularization through the abundant expression of CD34.

In summary, extensive p-CaSR/CaSR expression was initially demonstrated in vascular endothelial cells of free flaps affected by I/R injury, and inhibition of p-CaSR might become a novel therapeutic target for free flaps affected by I/R injury. The authors conclude that activation of the SDF-1*α*/CXCR4 axis and NPS2143 could protect free flaps from I/R injury and counteract partial or extensive CaSR activation-mediated necrosis of free flaps by inhibiting extensive caspase-3/caspase-9 expression-induced cell apoptosis and enhancement of MVD in the neovascularization of free flaps. However, the underlying mechanism of the interactions between the SDF-1*α*/CXCR4 axis and CaSR or other G protein-coupled receptors remain to be elucidated.

## Supplementary Materials

Supplementary Materials Supplemental Figure  1 A 3-h period of flap ischemia was elected among different time points (i.e., hours 1, 2, 3, and 4) because of the highest expression of p-CaSR protein. *∗∗∗P* < 0.001 versus 3-h of ischemia.
