# Correction to “Stromal Cell‐Derived Factor‐1*α* Alleviates Calcium‐Sensing Receptor Activation‐Mediated Ischemia/Reperfusion Injury by Inhibiting Caspase‐3/Caspase‐9‐Induced Cell Apoptosis in Rat Free Flaps”

**DOI:** 10.1155/bmri/9845949

**Published:** 2026-10-15

L. Song, L. Gao, J. Wang, et al. “Stromal Cell‐Derived Factor‐1*α* Alleviates Calcium‐Sensing Receptor Activation‐Mediated Ischemia/Reperfusion Injury by Inhibiting Caspase‐3/Caspase‐9‐Induced Cell Apoptosis in Rat Free Flaps,” *BioMed Research International*, 2018, https://doi.org/10.1155/2018/8945850.

In the article, the authors have identified errors in Figure 2A, caused by incorrect data being used in the preparation of the Figure. Specifically, the incorrect images were used in:

• The B:NPS2143 + CaCl_2_, 7d after I/R panel
• The C:SDF‐1*α* + CaCl_2_, 7d after I/R panel

Additionally, it was identified during further investigation that in Figure 3, the Cyt‐c/2 h after I/R bands are identical to the Bax/7d after I/R bands.

The correct Figures 2 and 3 are shown below:

**Figure 2:**
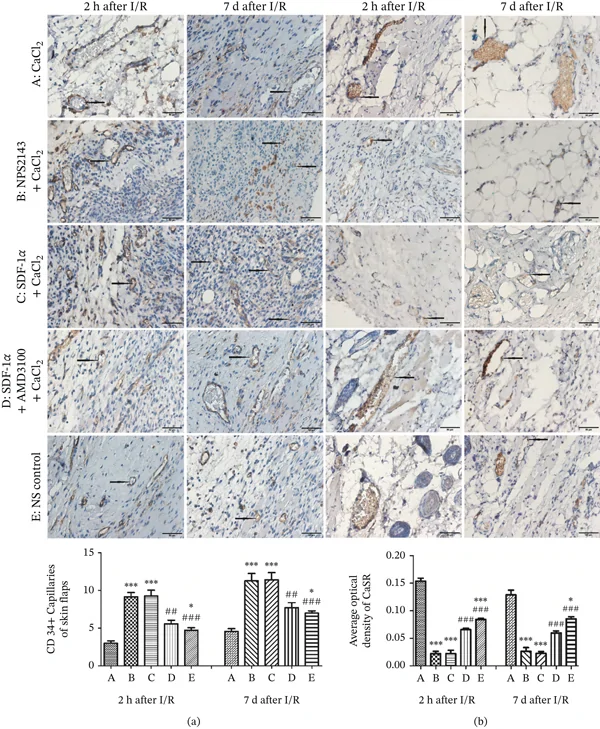
(a) MVD in neovascularization stained by CD34‐positive capillaries was examined using IHC staining after 2 h or 7 days of reperfusion. The CaCl2 group yielded the lowest MVD, whereas the NPS2143 + CaCl_2_ and SDF‐1*α* + CaCl_2_ groups exhibited notably enhanced neovascularization compared with the control group. (b) CaSR by IHC staining after 2 h or 7 days of reperfusion. The CaCl_2_ group exhibited the highest expression of CaSR in vascular endothelial cells and partial blood cells, whereas the expressions levels of CaSR in the NPS2143 + CaCl_2_ and SDF‐1*α* + CaCl_2_ groups were lower than those in the NS control group.  ^∗∗∗^ *p* < 0.001 versus CaCl_2_ group,  ^∗^ *p* < 0.05 versus CaCl_2_ group, ###*p* < 0.001 versus SDF‐1*α* + CaCl_2_ group, ##*p* < 0.01 versus SDF‐1*α* + CaCl_2_ group.

**Figure 3:**
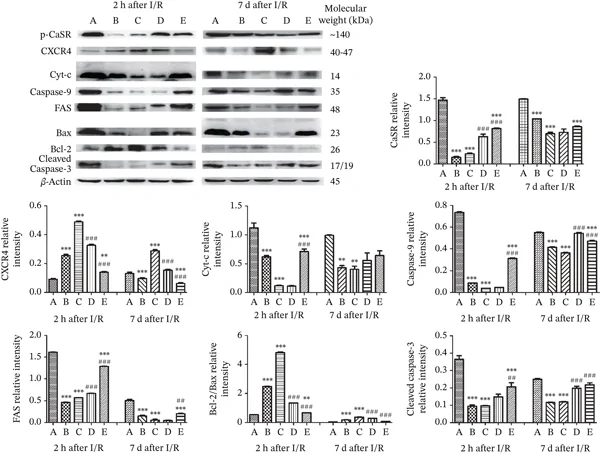
The protein expression levels of p‐CaSR, CXCR4, FAS, Cyt‐c, caspase‐9, Bax, Bcl‐2, and cleaved caspase‐3 were assessed by western blot analysis after 2 h or 7 days of reperfusion. The expression levels of p‐CaSR, FAS, Cyt‐c, caspase‐9, and Bax were the highest in the CaCl_2_ group, whereas the expression levels of these proteins were significantly lower in the NPS2143 + CaCl_2_ and SDF‐1*α* + CaCl_2_ groups. The expression levels of p‐CaSR showed a significant difference at Hour 2 but not on Day 7 after I/R of free flaps. The expression level of CXCR4 was the strongest, whereas that of caspase‐9 was the weakest in the SDF‐1*α* + CaCl_2_ group over time.  ^∗∗∗^ *p* < 0.001 versus CaCl_2_ group,  ^∗∗^ *p* < 0.01 versus CaCl_2_ group, ###*p* < 0.001 versus SDF‐1*α* + CaCl_2_ group, ##*p* < 0.01 versus SDF‐1*α* + CaCl_2_ group.

We apologize for these errors.

