# Supplementary material for: Stromal Cell-Derived Factor-1α Alleviates Calcium-Sensing Receptor Activation-Mediated Ischemia/Reperfusion Injury by Inhibiting Caspase-3/Caspase-9-Induced Cell Apoptosis in Rat Free Flaps
Source: Biomed Res Int. 2018 Jan 11;2018:8945850. doi: 10.1155/2018/8945850 (PMC5820583; doi:10.1155/2018/8945850)

Supplemental Figure 1 A 3-h period of flap ischemia was elected among different time points (i.e., hours 1, 2, 3, and 4) because of the highest expression of p-CaSR protein. ***P<0.001 versus 3-h of ischemia.

Supplemental Figure 1

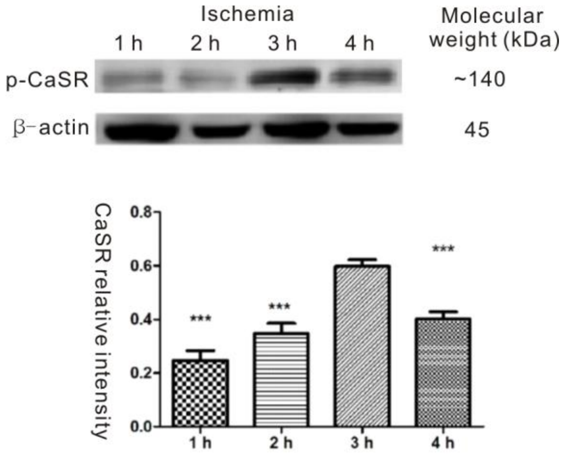

Supplement: Supplementary Materials — Supplemental Figure 1 A 3-h period of flap ischemia was elected among different time points (i.e., hours 1, 2, 3, and 4) because of the highest expression of p-CaSR protein. ∗∗∗P < 0.001 versus 3-h of ischemia. [file 8945850.f1.pdf]
